# Supplementary material for: Exposure to Green, Blue and Historic Environments and Mental Well-Being: A Comparison between Virtual Reality Head-Mounted Display and Flat Screen Exposure
Source: Int J Environ Res Public Health. 2022 Aug 2;19(15):9457. doi: 10.3390/ijerph19159457 (PMC9368727; doi:10.3390/ijerph19159457)
Supplement: Supplementary file 1 [file ijerph-19-09457-s001.zip › ijerph-1829301-supplementary.pdf]

Supplementary Materials

Table S1: Mean (SD) theta activity

		Overall Frontal	Overall Occipital	Medial Frontal	Lateral Frontal	Left Frontal	Right Frontal
**Green**	FS	1.75(0.63)	1.39(0.72)	1.01(0.34)	3.07(1.65)	2.25(1.19)	1.83(0.78)
	VR	3.07(1.25)	1.72(1.13)	1.82(1.31)	4.79(1.97)	3.33(1.36)	3.28(1.78)
**Blue**	FS	1.85(0.95)	1.19(0.67)	1.19(0.71)	3.22(2.26)	2.37(1.43)	2.03(1.34)
	VR	3.00(0.96)	1.70(1.62)	2.02(1.71)	5.20(1.92)	4.22(1.87)	3.00(1.17)
**Historic**	FS	1.89(0.71)	1.62(1.07)	1.11(0.45)	3.61(1.64)	2.50(1.21)	2.22(0.79)
	VR	3.84(1.47)	2.53(2.35)	2.22(1.99)	6.15(3.27)	4.11(1.44)	4.25(2.19)
**Traffic**	FS	2.29(0.78)	1.72(0.86)	1.29(0.68)	3.80(1.42)	2.73(1.02)	2.37(1.00)

Table S2: Mean (SD) alpha activity

		Overall Frontal	Overall Occipital	Medial Frontal	Lateral Frontal	Left Frontal	Right Frontal
**Green**	FS	0.78(0.38)	0.99(0.50)	0.61(0.31)	1.14(0.73)	1.03(0.77)	0.72(0.29)
	VR	1.19(0.57)	1.25(0.59)	0.74(0.38)	1.53(0.59)	1.18(0.43)	1.09(0.47)
**Blue**	FS	0.80(0.52)	1.12(1.00)	0.78(0.46)	1.26(0.88)	1.19(0.68)	0.79(0.45)
	VR	1.17(0.54)	1.38(0.76)	0.79(0.38)	1.60(0.65)	1.27(0.45)	1.12(0.51)
**Historic**	FS	0.76(0.43)	1.40(1.04)	0.64(0.41)	1.11(0.59)	0.95(0.61)	0.80(0.37)
	VR	1.31(0.67)	1.28(0.65)	0.85(0.56)	1.72(0.71)	1.34(0.56)	1.22(0.66)
**Traffic**	FS	1.08(0.56)	1.11(0.64)	0.72(0.45)	1.43(0.63)	1.15(0.55)	1.42(0.59)

Table S3: Mean (SD) beta activity

		**Overall Frontal**	**Overall Occipital**	**Medial Frontal**	**Lateral Frontal**	**Left Frontal**	**Right Frontal**
**Green**	FS	0.47(3.18)	0.41(0.31)	0.39(0.33)	0.59(0.51)	0.49(0.40)	0.50(0.50)
	VR	1.00(0.80)	0.86(0.71)	0.80(0.86)	1.07(0.91)	0.95(0.89)	0.92(0.79)
**Blue**	FS	0.81(1.24)	0.71(1.06)	0.68(1.37)	0.85(1.39)	0.85(1.58)	0.68(1.19)
	VR	0.76(0.48)	0.70(0.46)	0.60(0.58)	0.83(0.44)	0.75(0.54)	0.68(0.35)
**Historic**	FS	0.54(0.77)	0.63(0.79)	0.49(0.79)	0.67(0.79)	0.55(0.76)	0.61(0.80)
	VR	0.91(0.49)	0.78(0.40)	0.69(0.52)	0.96(0.57)	0.86(0.52)	0.80(0.42)
**Traffic**	FS	0.58(0.59)	0.57(0.84)	0.44(0.60)	0.74(0.69)	0.62(0.63)	0.56(0.62)

Table S4: Mean (SD) gamma activity

		**Overall Frontal**	**Overall Occipital**	**Medial Frontal**	**Lateral Frontal**	**Left Frontal**	**Right Frontal**
**Green**	FS	0.32(0.36)	0.26(0.48)	0.20(0.26)	0.58(1.00)	0.37(0.70)	0.42(0.93)
	VR	0.58(0.36)	0.48(0.47)	0.38(0.27)	0.68(0.43)	0.49(0.36)	0.57(0.33)
**Blue**	FS	0.34(0.50)	0.26(0.39)	0.18(0.29)	0.30(0.30)	0.24(0.34)	0.24(0.26)
	VR	0.50(0.34)	0.47(0.53)	0.32(0.21)	0.66(0.46)	0.51(0.39)	0.48(0.23)
**Historic**	FS	0.26(0.26)	0.25(0.33)	0.17(0.20)	0.49(0.59)	0.30(0.28)	0.36(0.47)
	VR	0.65(0.39)	0.55(0.47)	0.45(0.28)	0.73(0.49)	0.56(0.40)	0.62(0.32)
**Traffic**	FS	0.35(0.37)	0.31(0.68)	0.20(0.26)	0.67(0.95)	0.45(0.70)	0.41(0.58)
